# A Hair Ball Removed from the Stomach

**Published:** 1902-02-01

**Authors:** 


### A HAIR BALL REMOVED FROM THE STOMACH.

At a recent meeting of the Clinical Society, Dr. E. Percy Paton related a curious case in which a hair ball was found in, and removed from, the stomach of a child nine years of age. The patient was an intelligent girl, in whom a lump was accidentally discovered in the epigastric region. She was known to have eaten hair and other similar substances since she was three years old, and she had only been recently broken of the habit. Although there were no gastric symptoms a hair ball was diagnosed, and on opening the stomach a mass was found and extracted, consisting chiefly of the child's own hair. It was seven inches long, weighed seven and a quarter ounces, and had the general shape of the stomach. The child recovered without any bad symptoms.

